# Supplementary material for: Medium-term outcomes of sacral neuromodulation in patients with refractory overactive bladder: A retrospective single-institution study
Source: PLoS One. 2020 Jul 9;15(7):e0235961. doi: 10.1371/journal.pone.0235961 (PMC7347151; doi:10.1371/journal.pone.0235961)
Supplement: Attachment — Submitted filename: Response to Reviewers.doc [file pone.0235961.s002.doc]

**Additional Editor Comments (if provided):**

I acted as a reviewer for this manuscript as well as the Academic Editor. I would recommend that the paper be considered after revision with several caveats suggested by the two senior reviewers with experience in SNM technology and implantation. Recommended to alter the discussion and introduction to expand on the methods of efficacy in SNM for OAB and on comparative clinical alternatives along with an expansion on the importance of the placebo effect. The authors are congratulated on an impressive set of results in the medium-term in a well written style. I would also suggest that the follow-up is medium and not long-term.

MANY THANKS FOR YOUR VALUABLE FEEDBACK. WE HAVE ADDRESSED THE REVIEWERS’ COMMENTS IN THE REVISED MANUSCRIPT (PLEASE SEE OUR RESPONSES BELOW). WE HAVE ALSO CHANGED THE FOLLOW-UP DURATION FROM LONG-TERM TO MEDIUM-TERM THROUGHOUT THE MANUSCRIPT (WHERE APPLICABLE).

**Journal Requirements:**

When submitting your revision, we need you to address these additional requirements:

1. Please ensure that your manuscript meets PLOS ONE's style requirements, including those for file naming. The PLOS ONE style templates can be found at http://www.plosone.org/attachments/PLOSOne_formatting_sample_main_body.pdf and http://www.plosone.org/attachments/PLOSOne_formatting_sample_title_authors_affiliations.pdf

THANK YOU. WE HAVE DONE THE NEEDFUL.

2. We noticed you have some minor occurrence(s) of overlapping text with the following previous publication(s), which needs to be addressed:

https://doi.org/10.1007/s00192-017-3546-6

In your revision ensure you cite all your sources (including your own works), and quote or rephrase any duplicated text outside the Methods section. Further consideration is dependent on these concerns being addressed.

THANK YOU FOR POINTING THIS OUT. AS ADVISED, WE HAVE REPHRASED THE OVERLAPPING TEXT IN OUR REVISED MANUSCRIPT.

3. Thank you for stating the following in the Financial Disclosure section:

"The authors received no specific funding for this work."

We note that one or more of the authors are employed by a commercial company: 'Clin-Science Research Consulting'.

Please provide an amended Funding Statement declaring this commercial affiliation, as well as a statement regarding the Role of Funders in your study. If the funding organization did not play a role in the study design, data collection and analysis, decision to publish, or preparation of the manuscript and only provided financial support in the form of authors' salaries and/or research materials, please review your statements relating to the author contributions, and ensure you have specifically and accurately indicated the role(s) that these authors had in your study. You can update author roles in the Author Contributions section of the online submission form.

AD ADVISED, WE HAVE DECLARED THE COMMERCIAL AFFILIATION (CLIN-SCIENCE RESEARCH CONSULTING) FOR THE SECOND AUTHOR. THE AUTHOR CONTRIBUTION SECTION HAS ALSO BEEN UPDATED.

Please also include the following statement within your amended Funding Statement.

“The funder provided support in the form of salaries for authors [insert relevant initials], but did not have any additional role in the study design, data collection and analysis, decision to publish, or preparation of the manuscript. The specific roles of these authors are articulated in the ‘author contributions’ section.”

If your commercial affiliation did play a role in your study, please state and explain this role within your updated Funding Statement.

AS SUGGESTED, WE HAVE NOW INCLUDED THE AMENDED FUNDING STATEMENT IN THE COVER LETTER ACCOMPANYING THE REVISED MANUSCRIPT.

Please also provide an updated Competing Interests Statement declaring this commercial affiliation along with any other relevant declarations relating to employment, consultancy, patents, products in development, or marketed products, etc.

Within your Competing Interests Statement, please confirm that this commercial affiliation does not alter your adherence to all PLOS ONE policies on sharing data and materials by including the following statement: "This does not alter our adherence to PLOS ONE policies on sharing data and materials.” (as detailed online in our guide for authors http://journals.plos.org/plosone/s/competing-interests) . If this adherence statement is not accurate and there are restrictions on sharing of data and/or materials, please state these. Please note that we cannot proceed with consideration of your article until this information has been declared.

AS ADVISED, WE HAVE CONFIRMED IN THE COVER LETTER THAT THE COMMERCIAL AFFILIATION DOES NOT ALTER OUR ADHERENCE TO PLOS ONE POLICIES ON SHARING DATA AND MATERIALS.

* Please include both an updated Funding Statement and Competing Interests Statement in your cover letter. We will change the online submission form on your behalf.

Please know it is PLOS ONE policy for corresponding authors to declare, on behalf of all authors, all potential competing interests for the purposes of transparency. PLOS defines a competing interest as anything that interferes with, or could reasonably be perceived as interfering with, the full and objective presentation, peer review, editorial decision-making, or publication of research or non-research articles submitted to one of the journals. Competing interests can be financial or non-financial, professional, or personal. Competing interests can arise in relationship to an organization or another person. Please follow this link to our website for more details on competing interests: http://journals.plos.org/plosone/s/competing-interests

AS ADVISED, WE HAVE INCLUDED BOTH AN UPDATED FUNDING STATEMENT AND COMPETING INTERESTS STATEMENT IN OUR COVER LETTER. WE WOULD BE GRATEFUL IF YOU COULD PLEASE UPDATE THE ONLINE SUBMISSION FORM ON OUR BEHALF. THANK YOU.

**Review Comments to the Author**

**Reviewer #1:** 1. The subjective assessment of the patients by the physician : What was the relative score\scale of the various symptoms-frequency, urgency and UUI (how was the >50% improvement calculated) before, immediately at the end of the test period as compared to that at the follow up. Was there a change in the symptomatic expression?

THANK FOR THIS QUERY. SUCCESS WAS DEFINED AS >=50% IMPROVEMENT IN ANY CLINICAL PARAMETER AT THE LAST FOLLOW-UP BASED ON THE SUBJECTIVE ASSESSMENT OF PATIENT’S RESPONSE BY THE PHYSICIAN AS MENTIONED IN THE CLINICAL NOTE. IN OTHER WORDS, SUCCESS WAS CONSIDERED IF THE NUMBER OF URGENCY, FREQUENCY OR UUI EPISODES DROPPED BY HALF OR MORE, AS ASSESSED BY THE PHYSICIAN BASED ON PATIENT’S RESPONSE.

THE ABOVE HAS BEEN CLARIFIED IN THE METHODS SECTION OF THE REVISED MANUSCRIPT.

2. The above information may enable to remove the column "primary SNM indication: in table 1.

THANK YOU FOR THIS SUGGESTION. HOWEVER, WE WOULD LIKE TO REQUEST RETAINING THIS COLUMN IN THE TABLE BECAUSE IT DESCRIBES THAT ONLY 3 PATIENTS HAD URGENCY-FREQUENCY SYMPTOMS WITH NO INCONTINENCE (I.E. DRY OAB), WHEREAS 63 HAD URGENCY INCONTINENCE (I.E. WET OAB). OUT OF THE 63 WET OAB PATIENTS, 35 HAD MIXED URINARY INCONTINENCE WITH URGENCY PREDOMINANCE AND 28 HAD URGENCY URINARY INCONTINENCE.

3. Since there are no significant factors predicting the success rate, Table 2 can be removed leaving only the reported results in writing.

WE UNDERSTAND THAT NONE OF THE EVALUATED FACTORS WERE SIGNIFICANT IN PREDICTING THE SUCCESS, HOWEVER WE WOULD STILL REQUEST TO RETAIN TABLE 2 IN THE MANUSCRIPT, BECAUSE THE TABLE PROVIDES THE PARAMETER ESTIMATES (OR ODDS RATIOS) FOR DIFFERENT RISK FACTORS WHICH MIGHT BE HELPFUL FOR OTHER RESEARCHERS IN THEIR FUTURE INVESTIGATIONS.

4. Complications: Pain (3) and probably decrease device efficacy (5) should be consider as complication thus raising the complication rate to 13\55=23.6%

AS SUGGESTED, WE HAVE INCLUDED PAIN AS A COMPLICATION, SO THE NEW COMPLICATION RATE IS NOW 8/55=14.5%. THIS HAS BEEN UPDATED THROUGHOUT THE MANUSCRIPT.

DECREASED DEVICE EFFICACY (N=5) WAS NOT A DIRECT COMPLICATION OF THE IMPLANT PROCEDURE AND CAN PARTIALLY BE CONTRIBUTED BY A PHYSIOLOGIC EFFECT DUE TO HABITUATION OF THE NERVOUS SYSTEM. AS A RESULT, WE HAVE NOT CONSIDERED DECREASED DEVICE EFFICACY AS A COMPLICATION.

**Reviewer #2**: The authors present a retrospective analysis of the efficacy of SNM in 66 consecutively managed OAB cases with excellent results of improvement in pad use in about ¾ cases over a medium-term follow-up period. There is a moderately high SNM revision rate. Analysis did not reveal factors predictive of efficacy.

I have several caveats:

1. Expand a little on the specialist groups in society affected by OAB and expand on the economic community burden in investigation and management.

AS SUGGESTED, THIS DESCRIPTION HAS NOW BEEN ADDED IN THE INTRODUCTION SECTION OF THE REVISED MANUSCRIPT. APPROPRIATE REFERENCES HAVE ALSO BEEN ADDED.

2. The authors need to separate the reported incidence of occasional incontinence in women which in some studies is exceptionally high. i.e. to clarify a little their definitions in the introduction.

AS ADVISED, WE HAVE ADDED MORE CLARITY ON THE DIFFERENT TYPES OF URINARY INCONTINENCE IN THE INTRODUCTION SECTION. PLEASE NOTE THAT OUR STUDY WAS FOCUSED ON PATIENTS WITH REFRACTORY OAB.

3. The inclusion of bladder augmentation and urinary diversion is a bit extreme and should be removed in my view.

AS ADVISED, WE HAVE REMOVED THESE TREATMENT OPTIONS FROM THE INTRODUCTION.

4. Can they briefly elaborate on the proposed mechanisms of SNM in OAB?

AS ADVISED, THIS HAS NOW BEEN DONE IN THE INTRODUCTION SECTION OF THE REVISED MANUSCRIPT. APPROPRIATE REFERENCES HAVE ALSO BEEN ADDED.

5. Comments on funding at the end of the discussion should be placed in a funding/financial disclosure statement and not at this point.

AS SUGGESTED, WE HAVE REMOVED THE FUNDING STATEMENT FROM THE DISCUSSION SECTION. AS A PART OF THE JOURNAL REQUIREMENT, WE HAVE NOW INCLUDED THE AMENDED FUNDING STATEMENT IN THE COVER LETTER ACCOMPANYING THE REVISED MANUSCRIPT.

6. Do we know anything about the clinical examination of these cases? Cystocele, rectocele, vault prolapse etc? I appreciate that these findings had no effect on efficacy.

THE FOLLOWING CONCOMITANT CONDITIONS WERE EVALUATED: VOIDING DYSFUNCTION, STRESS URINARY INCONTINENCE [SUI], PELVIC ORGAN PROLAPSE, RECURRENT URINARY TRACT INFECTION [UTI], AND FECAL INCONTINENCE. WITHIN THE CATEGORY OF PELVIC ORGAN PROLAPSE, THE FOLLOWING CONDITIONS WERE INCLUDED: CYSTOCELE, ENTEROCELE, RECTOCELE, UTERINE PROLAPSE AND VAGINAL VAULT PROLAPSE. THIS HAS NOW BEEN CLARIFIED IN THE METHODS SECTION OF THE REVISED MANUSCRIPT.

7. The authors discuss MRI compatible implantation…they could expand on some of the rechargeable devices which have been introduced for SNM with a conditional safety for full body MRI (e.g. De Wachter et al 2019) although it is accepted that these are less a requirement for some OAB cases and more for those cases needing high battery life. At any rate there can be a little extra discussion here.

AS ADVISED, WE HAVE NOW INCLUDED SOME DISCUSSION AROUND THIS NEW DEVICE IN THE REVISED MANUSCRIPT. APPROPRIATE REFERENCE HAS ALSO BEEN ADDED.

8. I would expand in the discussion on the proposed mechanisms of action of SNM in OAB (see above). I would also extend the level of sophistication of the discussion to expand on alternatives with comparison and consideration of their role e.g. PTNS, Transcutaneous TNS, peripheral implantables, transvaginal electrostimulation.

AS ADVISED, WE HAVE NOW EXPANDED THE DISCUSSION ON THE ROLE OF SOME TREATMENT ALTERNATIVES FOR OAB (ALONG WITH THE SUPPORTING REFERENCES).

9. Some comment needs to be made regarding the placebo effect and its comparison with other forms of stimulation e.g. the SuMit Trial and others…these need to be referenced in my opinion

AS ADVISED, A DISCUSSION ON PACEBO EFFECT (ALONG WITH SUMIT TRIAL AND OTHER RELEVANT STUDIES) HAS BEEN INCLUDED IN THE REVISED MANUSCRIPT.

Overall, I enjoyed this well written article. The data are limited but impressive and the article is short and punchy with a good design. I have added some caveats to consider in a revision of this useful manuscript.

I appreciate the opportunity of reviewing this interesting article.

THANK YOU FOR ALL YOUR CONSTRUCTIVE FEEDBACK IN HELPING US IMPROVE OUR MANUSCRIPT.
