# Supplementary material for: Medium-term outcomes of sacral neuromodulation in patients with refractory overactive bladder: A retrospective single-institution study
Source: PLoS One. 2020 Jul 9;15(7):e0235961. doi: 10.1371/journal.pone.0235961 (PMC7347151; doi:10.1371/journal.pone.0235961)
Supplement: Attachment — Submitted filename: Response to Reviewers_June 06 2020.doc [file pone.0235961.s003.doc]

ATTACHED BELOW, FOR YOUR PERUSAL, IS A DETAILED DESCRIPTION (HIGHLIGHTED IN RED AND CAPS) OF HOW WE’VE ADDRESSED THE REVIEWERS’ COMMENTS.

**Additional Editor Comments:**

I take this opportunity to point out to you that the origin of 'overactive bladder' in the ICS is not overactive bladder, but 'overactive bladder syndrome' and ask you to correct this.

THANKS FOR POINTING THIS OUT. AS SUGGESTED, WE HAVE MADE THIS CORRECTION IN THE REVISED MANUSCRIPT (BOTH IN THE ABSTRACT AND THE MAIN TEXT).

You have also introduced 'voiding dysfunction' and 'SUI' in the manuscript. Your manuscript does however not state that it has been established other than on the basis of symptoms. That is why I ask you to think about replacing with 'voiding symptoms' and 'symptoms of stress urinary incontinence'.

WE HAVE MADE THE SUGGESTED REPLACEMENTS THROUGHOUT THE MANUSCRIPT.

You also seem, as a reaction to earlier reviews, to be concerned about the numbers of patients with the syndrome and the costs involved, I agree with that. Can you include in the discussion how your approach - with an expensive intervention, without any kind of objective diagnosis - can lead to a reduction in costs and whether, for example, how medical self-regulation can be used to reduce costs?

THE FOCUS OF OUR PAPER WAS NOT ON THE COST-BENEFIT ANALYSIS OF SNM, HOWEVER, BASED ON YOUR SUGGESTION, WE HAVE INCLUDED A GENERAL POINT IN THE DISCUSSION THAT THE LONG-TERM COST BENEFITS OF SNM NEED TO BE INVESTIGATED IN FUTURE STUDIES.

**Review Comments to the Author**

**Reviewer #1:** The authors have answered the reviewers’ comments. It is a detailed though retrospective work which reassures physicians as well as patients with ORB to use SNM as a sound therapeutic option. The work is now eligible for publication.

MANY THANKS ONCE AGAIN FOR YOUR VALUABLE FEEDBACK.

**Reviewer #3:** The authors have a nice retrospective case series with 66 patients having a median follow up of 32 months. This is not long-term but they have had excellent outcomes with 74.5% reporting >50% improvement.

THANK YOU.

They report 27.3% explant at 24 months. This is high, even for 32 months’ follow up, when compared to 19.1% by Siegel et al who report 5 year outcomes for a multicenter RCT (INSITE)[1]. It should be clarified by the authors how many of these 27% got re-implanted when appropriate and for which reasons. NONE OF THESE 27% (N=15) PATIENTS GOT RE-IMPLANTED. THIS HAS BEEN CLARIFIED IN THE RESULTS SECTION OF THE REVISED MANUSCRIPT. The 5 explanted for loss of efficacy should be specifically stated for the reader if they were counted as therapeutic failures. YES, THEY WERE CONSIDERED THERAPEUTIC FAILURES. THIS HAS BEEN CLARIFIED IN THE RESULTS SECTION OF THE REVISED MANUSCRIPT.

The authors make the statement that medium and long term outcomes for safety and efficacy for SNM are limited. This is not really supported by the literature [1-9] with outcomes as long as 17 years for tined lead removal [6] and one manuscript dating back to 2010 with 5 year average outcomes [5].

THANK YOU FOR BRINGING THESE ADDITIONAL REFERENCES TO OUR ATTENTION. WE HAVE NOW INCLUDED A FEW OF THOSE IN THE INTRODUCTION SECTION AND HAVE ALSO MODIFIED THE STATEMENT REGARDING LIMITED DATA.

PLOS One requires that all data be made available to the reader, it is not part of the submitted manuscript and as far as I can tell, not submitted as supporting information. This should be remedied as a requirement of the journal.

THANKS FOR RAISING THIS POINT. PLEASE NOTE THAT AS PER THE JOURNAL REQUIREMENTS, WE HAVE INCLUDED THE FOLLOWING IN OUR COVER LETTER TO THE EDITOR.

We would like to confirm that the data underlying the findings in our study cannot be made publicly available due to ethical and legal restrictions. The original data contains potentially identifying or sensitive patient information. This restriction has been imposed by the Allen College Institutional Review Board (ACIRB). However, a copy of the de-identified dataset underlying the analyses reported in this paper is available to all interested researchers upon request to the ACIRB (contact person is Lisa Brodersen who can be reached at Lisa.Brodersen@allencollege.edu or 319-226-2000).

Table 1, it is a little confusing that the authors report mean followed by a comma, then median results. This would normally be reported as 2 separate columns and I suspect that will be an easy change and make it easier for the reader to understand

AS ADVISED, WE HAVE REPORTED MEANS AND MEDIANS IN 2 SEPARATE COLUMNS.

Table 2, for what it’s worth I agree with the authors’ response to reviewer #1 that although no factors were significant predictors, calling attention to this with a table may be helpful to some readers. I think publishing negative results can be nearly as helpful as positive ones in some cases.

WE AGREE WITH YOUR THOUGHTS ON THIS. AS A RESULT, WE HAVE RETAINED TABLE 2 IN OUR REVISED MANUSCRIPT.

Overall this appears to be a community based medium-term case series and may be of interest to some readers for that reason.

THANKS AGAIN FOR TAKING THE TIME TO REVIEW OUR MANUSCIPT AND PROVIDING YOUR VALUABLE COMMENTS.

[1] Siegel S, Noblett K, Mangel J, Bennett J, Griebling TL, Sutherland SE, et al. Five-Year Followup Results of a Prospective, Multicenter Study of Patients with Overactive Bladder Treated with Sacral Neuromodulation. J Urol. 2018;199:229-36.

[2] Amundsen CL, Komesu YM, Chermansky C, Gregory WT, Myers DL, Honeycutt EF, et al. Two-Year Outcomes of Sacral Neuromodulation Versus OnabotulinumtoxinA for Refractory Urgency Urinary Incontinence: A Randomized Trial. Eur Urol. 2018;74:66-73.

[3] Blok B, Van Kerrebroeck P, de Wachter S, Ruffion A, Van der Aa F, Perrouin-Verbe MA, et al. Two-year safety and efficacy outcomes for the treatment of overactive bladder using a long-lived rechargeable sacral neuromodulation system. Neurourol Urodyn. 2020.

[4] Ismail S, Chartier-Kastler E, Perrouin-Verbe MA, Rose-Dite-Modestine J, Denys P, Phe V. Long-Term Functional Outcomes of S3 Sacral Neuromodulation for the Treatment of Idiopathic Overactive Bladder. Neuromodulation. 2017;20:825-9.

[5] Marinkovic SP, Gillen LM, Marinkovic CM. Minimum 6-year outcomes for interstitial cystitis treated with sacral neuromodulation. Int Urogynecol J. 2011;22:407-12.

[6] Powell CR, Kreder KJ. Long-term outcomes of urgency-frequency syndrome due to painful bladder syndrome treated with sacral neuromodulation and analysis of failures. J Urol. 2010;183:173-6.

[7] Rueb JJ, Pizarro-Berdichevsky J, Goldman HB. 17-Year Single Center Retrospective Review of Rate, Risk Factors, and Outcomes of Lead Breakage during Sacral Neuromodulation Lead Removal. J Urol. 2020:101097JU0000000000000740.

[8] Yazdany T, Bhatia N, Nguyen J. Determining outcomes, adverse events, and predictors of success after sacral neuromodulation for lower urinary disorders in women. Int Urogynecol J. 2011;22:1549-54.

[9] Zegrea A, Kirss J, Pinta T, Rautio T, Varpe P, Kairaluoma M, et al. Outcomes of sacral neuromodulation for chronic pelvic pain: a Finnish national multicenter study. Tech Coloproctol. 2020;24:215-20.
