# Supplementary material for: Medium-term outcomes of sacral neuromodulation in patients with refractory overactive bladder: A retrospective single-institution study
Source: PLoS One. 2020 Jul 9;15(7):e0235961. doi: 10.1371/journal.pone.0235961 (PMC7347151; doi:10.1371/journal.pone.0235961)
Supplement: Attachment — Submitted filename: Response to Reviewers_June 23 2020.doc [file pone.0235961.s004.doc]

ATTACHED BELOW, FOR YOUR PERUSAL, IS A DETAILED DESCRIPTION (HIGHLIGHTED IN RED AND CAPS) OF HOW WE’VE ADDRESSED THE REVIEW COMMENTS.

**Additional Editor Comments:**

This area is saturated in the literature and the work provides not much news. It is not easy to decide positive for publication if you do not make the data publicly available, which is a PLOS ONE requirement. When there are ethical considerations, they are not different when data is available only 'on request'.

AS SUGGESTED, WE HAVE MADE THE DATA PUBLICLY AVAILABLE. A COPY OF THE DE-IDENTIFIED DATASET IN MS EXCEL FORMAT HAS BEEN UPLOADED TO THE SUBMISSION SYSTEM.
